# Study of nanostructure growth with nanoscale apex induced by femtosecond laser irradiation at megahertz repetition rate

**DOI:** 10.1186/1556-276X-8-185

**Published:** 2013-04-22

**Authors:** Nikunj B Patel, Bo Tan, Krishnan Venkatakrishnan

**Affiliations:** 1 Department of Aerospace Engineering, Ryerson University, Victoria Street, Toronto, ON M5B 2K3, Canada; 2 Department of Mechanical and Industrial Engineering, Ryerson University, Victoria Street, Toronto, ON M5B 2K3, Canada

**Keywords:** Laser material processing, Leaf-like nanostructures, Nanostructure growth mechanism

## Abstract

Leaf-like nanostructures with nanoscale apex are induced on dielectric target surfaces by high-repetition-rate femtosecond laser irradiation in ambient conditions. We have recently developed this unique technique to grow leaf-like nanostructures with such interesting geometry without the use of any catalyst. It was found to be possible only in the presence of background nitrogen gas flow. In this synthesis method, the target serves as the source for building material as well as the substrate upon which these nanostructures can grow. In our investigation, it was found that there are three possible kinds of nanotips that can grow on target surfaces. In this report, we have presented the study of the growth mechanisms of such leaf-like nanostructures under various conditions such as different laser pulse widths, pulse repetition rates, dwell times, and laser polarizations. We observed a clear transformation in the kind of nanotips that grew for the given laser conditions.

## Background

Nanostructures with nanoscale apex have become the center of attraction for many researchers around the world. These nanostructures have been widely named as nanotips, nanocones, nanonails, nanopencils, nanojets, and nanoneedles. They are considered to be one-dimensional nanostructures with a significantly large surface-to-volume ratio which is very desirable for the development of various novel devices. These nanostructures provide unique optical, electronic, mechanical, chemical, and other properties that can be very useful for the improvement of electronics interconnects, scanning probes, nanoelectronics, photovoltaic devices, electron field microemitters, light-emitting diodes, and photo-detectors [1,2]. Until now, such nanostructures have been mainly generated from materials such as ZnO, AlN, single and polycrystalline silicon, gold, and carbon whose growth is dependent on the crystallographic orientation. These nanostructures have been synthesized by techniques such as thermal evaporation, various types of chemical vapor deposition, resonance plasma etching, and chemical etching [2-8]. The aforementioned techniques require a long processing time, multiple steps, catalyst-assisted growth, high processing temperatures, very sophisticated equipment, vacuum, and clean room operations.

In the past few years, various types of lasers have also been utilized to produce micronanostructures with sharp ends (nanobumps, nanojets, nanoprotrusions) from the irradiation of thin metal films and bulk materials using tightly focused laser beams. Such sharp nanojet structures have been produced on gold thin films by irradiation of single nano- or femtosecond laser pulse in ambient or under low-vacuum conditions using circular laser spots [9]. In most of these cases, the gold films with certain thicknesses were deposited onto borosilicate glass or single-crystal silicon substrates by RF sputtering with the help of *in situ* coating of adhesion layers [9,10]. In these techniques, for each laser pulse interaction with the film, only one nanostructure is produced at a time, and the distance between two laser incident spots on the film has to be maintained at a certain value to avoid potential rupture of the film and the damage of the previously formed nanostructure via intersection of laser irradiation spots [11]. This eventually limits the number of nanostructures that can be produced on a surface area of the target. The study of these nanostructures for various parameters has been conducted by various researchers on various metal films [9-12]. The number of laser pulses that can be applied onto a particular spot on the target film is limited due to the fact that multiple laser pulses could ablate all the film material from the irradiation spot and could eventually start ablating the substrate surface. However, the multiple laser pulses have been used to produce sharp spikes on bulk silicon surfaces in vacuum chamber filled with 500 Torr of Cl_2_, SF_6_, N_2_, or He gas [13]. They have reported that the silicon surface irradiated in SF_6_ and Cl_2_ gas background exhibits the growth of sharp spikes roughly aligned in rows whereas in the case of vacuum, N_2_, or He gas background, very blunt spikes with irregular sides and rounded tops with much larger tip diameter are formed. The chemical reaction between silicon surface and the surrounding gas has been suggested to be the driving factor for the formation of this sharp spikes, although the exact formation mechanism is still unknown [13]. In another investigation, the silicon spikes have also been produced by femtosecond laser irradiation in submerged condition in water [14]. The spikes produced in this method are one to two orders of magnitude smaller than spikes induced in [13]. The silicon wafer is placed in a glass container filled with distilled water which is mounted on a three-axis translation stage. In their investigation, they found that for each incident laser pulse onto the silicon surface, two to three microbubbles are created in the water corresponding to which the same number of ripple-like structures are created onto the silicon surface. As more laser pulses are applied, more numbers of ripple structures are created which start to overlap with each other and roughens the silicon surface. These interactions result in generation of many submicrometer bead-like structures on silicon surface which eventually sharpen and grow into spikes through preferential removal of material around the beads by laser-assisted etching.

**Figure 1:**
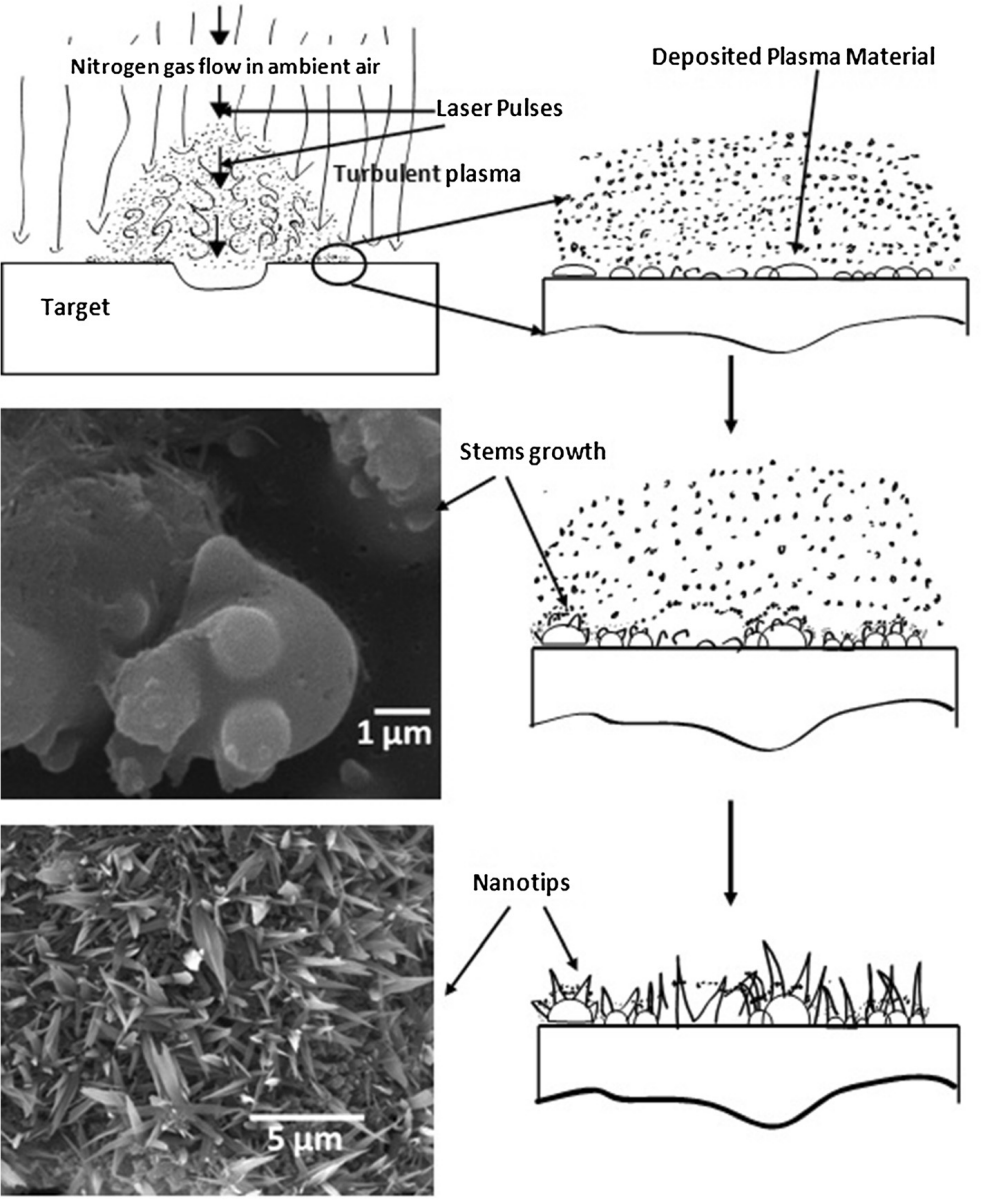
**Nanotip growth.** Schematic representation of our femtosecond laser pulses that induced nanotip growth process with supporting SEM images.

Recently, our research group developed a unique technique to produce leaf-like nanotips utilizing the interaction of femtosecond laser-generated plasma from target transparent glass with nitrogen gas flow background under ambient conditions [15]. Some of the benefits of our method in comparison to the aforementioned techniques include that it allows us to generate nanotips from amorphous dielectric material which, to our best knowledge, has never been attempted before, and it is a catalyst-free growth mechanism. The process is performed in open air at ambient conditions under nitrogen gas flow. In this very simple and rapid technique, the target behaves as the source to provide building material for nanostructure growth as well as substrate upon which these unique nanostructures can grow, as depicted in Figure 1. High-energy plasma is generated when the target is irradiated with laser pulses at megahertz repetition rate. This plasma expands outward and interacts with nitrogen gas and incoming laser pulses. The vapor condensates from the plasma continuously get deposited back to the target surface, as depicted in Figure 1. This deposited material experience a variable amount of internal and external pressure because of the difference of the temperature between the target surface, the plasma, and surrounding air, and also variable cooling due to nitrogen gas flow. These force variations on deposited material initiate the stems’ growth upon which the subsequent plasma condensates get deposited and form leaf-like nanotip structures with nanoscale apex, as shown in Figure 1 schematics and scanning electron microscopy (SEM) images.

In this paper, we report the study of how the formation of leaf-like nanotips on femtosecond laser-irradiated transparent dielectric target is influenced due to various femtosecond laser pulse widths, repetition rates, machining dwell time, and laser polarization. Understanding the changes in generated nanotips will help us pick the right combinations of laser parameters to grow the desired amount and kind of nanotips over the large surface area of dielectric targets.

## Methods

The experiments were performed on plain microscopic slide glass with composition of 60% to 75 wt.% SiO_2_, 5% to 12 wt.% CaO, and 12% to 18 wt.% Na_2_O. A direct-diode-pumped Yb-doped fiber amplifier/oscillator system (wavelength, *λ* = 1,030 nm) capable of delivering a maximum average output of 16 W was used as a femtosecond laser source to irradiate targets with thickness of 0.90 to 1.0 mm. The laser intensity profile beam was focused into a spot (full width at half-maximum) diameter of 10 μm on the target surface using a telecentric lens of 100-mm effective focal length. The same setup was used to perform these experiments as reported in a previous paper done by our research group [16]. However, for these experiments, a square bracket was placed in front of the target surface which holds six nozzles providing continuous flow of nitrogen gas. The machining was performed in the form of 26 × 26 arrays of microholes for various femtosecond laser parameters. We investigated the effect of three different pulse widths (214, 428, and 714 fs) on the generation of nanotips for a repetition rate of 13 MHz at a dwell time of 0.5 ms. The effect of various laser pulse repetition rates (4, 8, and 13 MHz) and different dwell times was also investigated on glass samples. All the aforementioned experiments were done by circular polarization of laser pulses. We also examined how different (linear, p-) polarizations would change the growth of nanotips on the target surface. The linear (p-) polarization of the beam was achieved by placing a half-wave plate in front of the focusing lens. The laser-irradiated glass samples have been analyzed by SEM.

## Results and discussion

It is found that laser conditions have great effect on the nanotip growth. They control the population and the shape of the synthesized nanotips. Table 1 summarizes the observations.

**Table 1:** Summary of effects of laser conditions to tip growth

**Laser parameters**	**Effects on nanotip growth**
Pulse width	Short pulses yield narrow long tips
Repetition rate	Higher repetition rate promotes the growth of dense, oriented narrow nanotips
Dwell time	Longer dwell time increases the population of nanotips. However, beyond an optimum dwell time, over heating will remelt the newly formed nanotips
Polarization	Linear (p-) polarization increases the population of nanotips

### Effect of pulse width

There are two mechanisms responsible for laser-induced optical breakdown of materials: multiphoton absorption and avalanche ionization. Multiphoton absorption results when a molecule absorbs the required amount of photons simultaneously to get ionized, which has proven to be the main mechanism for breakdown in the low-femtosecond regime [17]. In our experiments, the investigated pulse widths fall above the low-femtosecond regime where the combination of both mechanisms is believed to be responsible for the breakdown. Multiphoton ionization is responsible for the initial generation of electrons which are further heated by incoming portion of the pulse resulting in avalanche ionization and rapid plasma formation [18]. The initial part of the pulse produces free-electron plasma which can absorb the later part more efficiently and/or behave as a mirror and reflect most of the incident energy [17,19,20]. Every material has its unique optical damage fluence, but all the pure dielectrics demonstrate similar behavior in all ranges of pulse width as observed for SiO_2_[21]. Stuart et al. investigated the threshold fluence for fused silica and CaF_2_ with laser pulses in the range 270 fs ≤ *τ* ≤ 1 ns [21]. They discovered that the damage threshold decreased with the decrease of the pulse width.

Fan and Longtin developed a femtosecond breakdown model which gives the time at which the laser intensity reaches the breakdown threshold at a given position [17], *T*_B_ (*Z*).

(1) $$T_{B}\left(Z\right)=\frac{Z}{C}\pm \tau_{p}\;\sqrt{\frac{1}{4\ln2}\ln\left[\beta {\left(1+\frac{Z^{2}}{Z_{R}^{2}}\right)}^{-1}\right]},$$

where *Z* is the axial location in the focal region (*Z* = 0 at focal point), *τ*_p_ is the full width at half-maximum pulse duration, *c* is the speed of light in a medium, *β* is the ratio of peak pulse power to the breakdown threshold of a material (*P*_max_/*P*_th_), and *Z*_R_ is the Rayleigh range or focal region,

$$Z_{R}=\mathrm{n\pi}\omega_{0}^{2}/\lambda .$$

**Figure 2:**
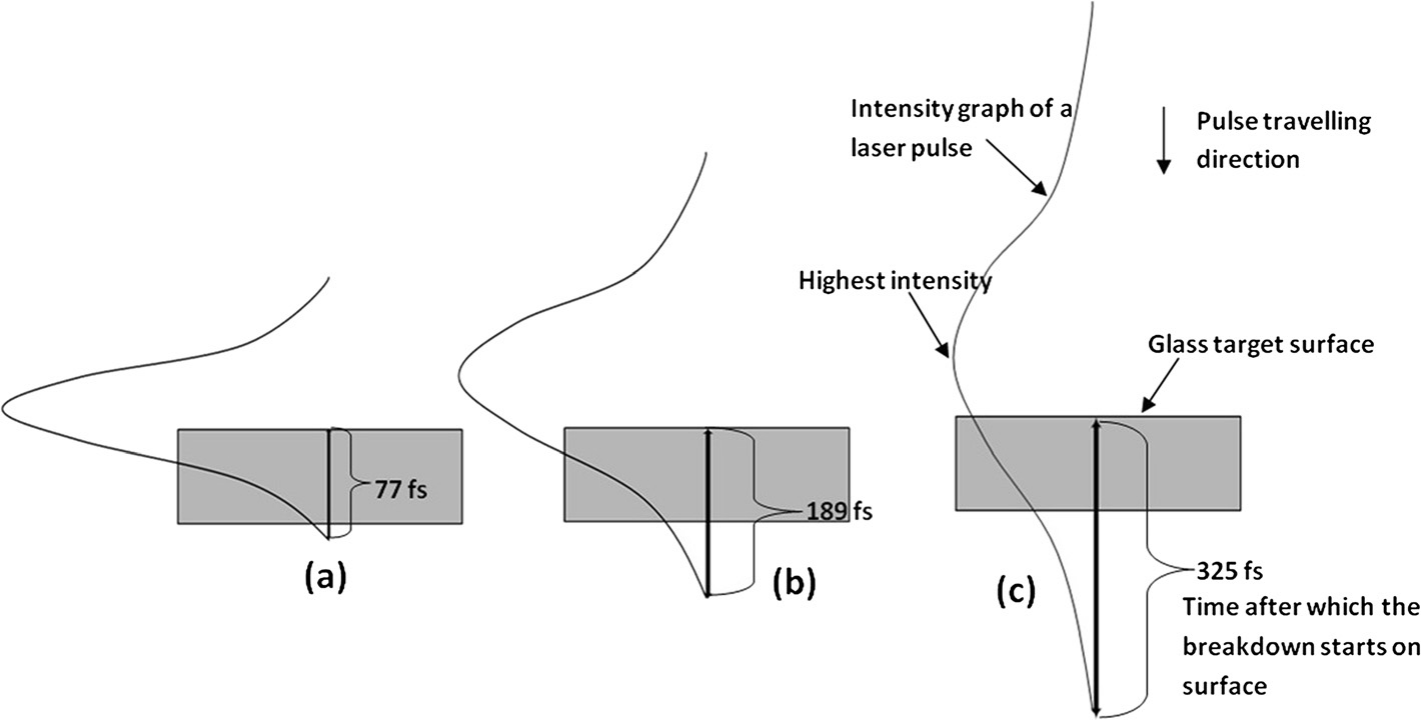
**Interaction of femtosecond laser pulses of different pulse-width sizes with glass surface.** Schematic representation of glass irradiation with femtosecond laser pulses with pulse widths of (**a**) 214, (**b**) 428, and (**c**) 714 fs (schematic not to the scale).

**Figure 3:**
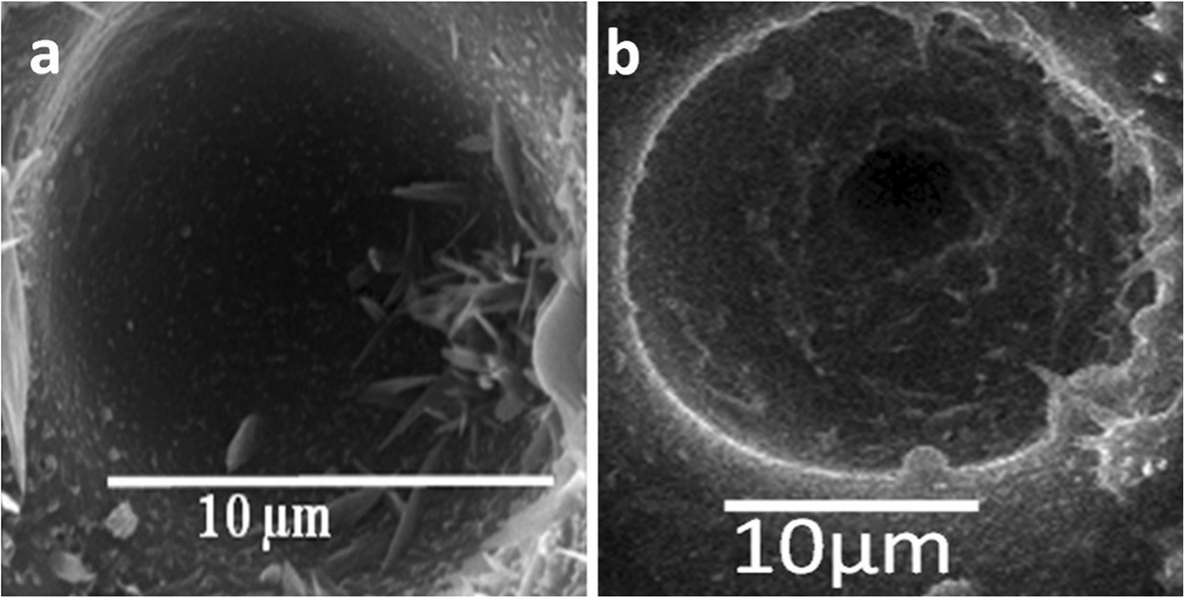
**Microholes drilled via different pulse-width sizes.** Microholes drilled by femtosecond laser pulses with pulse widths of (**a**) 214 and (**b**) 714 fs at 16-W average laser power and 0.5-ms dwell time, 13-MHz repetition rate.

Equation 1 gives the time at which the breakdown starts after the laser pulse has started interacting with the target surface at a given position in the focal region. From this point onward, the plasma starts to grow and expand, and covers the irradiated spot for few nanoseconds during which the second part of the laser pulse is still traveling toward the target surface. Using this equation, the time required for the breakdown to initiate is calculated to be 77, 189, and 325 fs for pulse widths of 214, 428, and 714 fs, respectively. The schematic representation of this time is shown in Figure 2. The amount of energy lost to the plasma before reaching the target surface depends on the amount of time the remaining portion, after breakdown initiation, of the pulse spends on traveling through the plasma. Shorter laser pulses (214 fs) reach threshold fluence very early since they possess high intensity, as depicted in Figure 2. However, they are very short and thus spend less amount of time in the plasma and thus loose less energy to the plasma and remove target material more efficiently compared to longer pulses (>214 fs). Hence, as can been seen from Figure 3a, the hole (approximately 12 μm in diameter) drilled by 214-fs pulse is closer in size to the laser beam spot diameter of 10 μm. Although we just worked with pulses in femtosecond regime (214 to 714 fs), the findings in the investigation by Stuart et al. are more relevant to our experiments as they worked with multiple pulses of laser with the wavelength of 1,053 nm in order to measure the damage threshold for SiO_2_[21]. Whatever results Stuart et al. achieved between picosecond and femtosecond pulses, we acquired it within the femtosecond pulse regime. For example, they discovered that the damage area generated by the 500-fs pulse in fused silica glass was twice as much smaller than that produced by the 900-ps pulse.

Even though we did not work in the picosecond pulse duration regime, we obtained similar result as we increased the pulse width in the femtosecond regime. Figure 3 shows the SEM images of the microholes drilled by femtosecond laser pulses at 13-MHz repetition rate for 0.5-ms dwell time with pulse widths of 214 and 714 fs, respectively. The diameters of these microholes are approximately on average 12 and 21 μm, respectively. The size of microhole represent the amount of material removed from the target; larger diameter means larger amount of material removal compare to smaller hole diameter. The life span of the plasma is also an important factor. In the current investigation, the turbulence created in the plasma due to the interactions between nitrogen gas and plasma species lengthens the plasma life. Since the longer pulses spend a significant portion of their duration traveling through previously formed plasma, as depicted in Figure 2, the energy transmitted via longer pulse is not enough to ablate the material upon contact with the target material. Rather, this transmitted energy gets stored in the top part of the lattice and gets transferred into the bulk in all directions, making the target temperature rise in the area surrounding the irradiated spot. This makes molecules to become loose to form a larger pool of molten material. As a result, the subsequent longer pulses expel large particles and droplets into the plasma upon contacting the molten pool. On the contrary, the interaction of the short pulses with the target surface does not rise as much high temperature which creates shallow molten pool. Hence, the material removed from the target is composed of smaller particles and droplets. The size of the plasma species and the temperature rise of the target surface greatly affect the type of nanotips that grow on the target surface.

**Figure 4:**
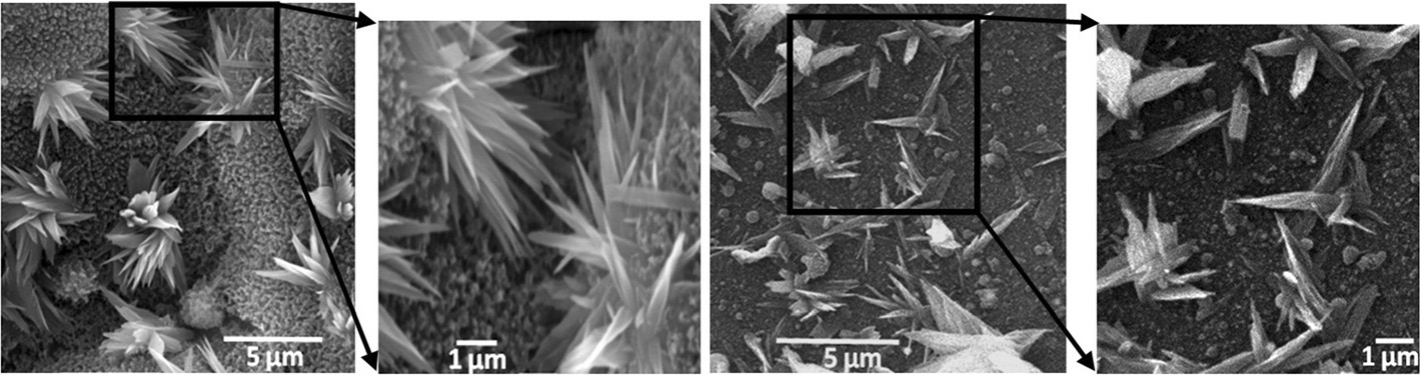
**Various types of nanotips.** Tips generated at 214 fs for 13 MHz at dwell time of 0.5 ms and 16-W average laser power.

**Figure 5:**
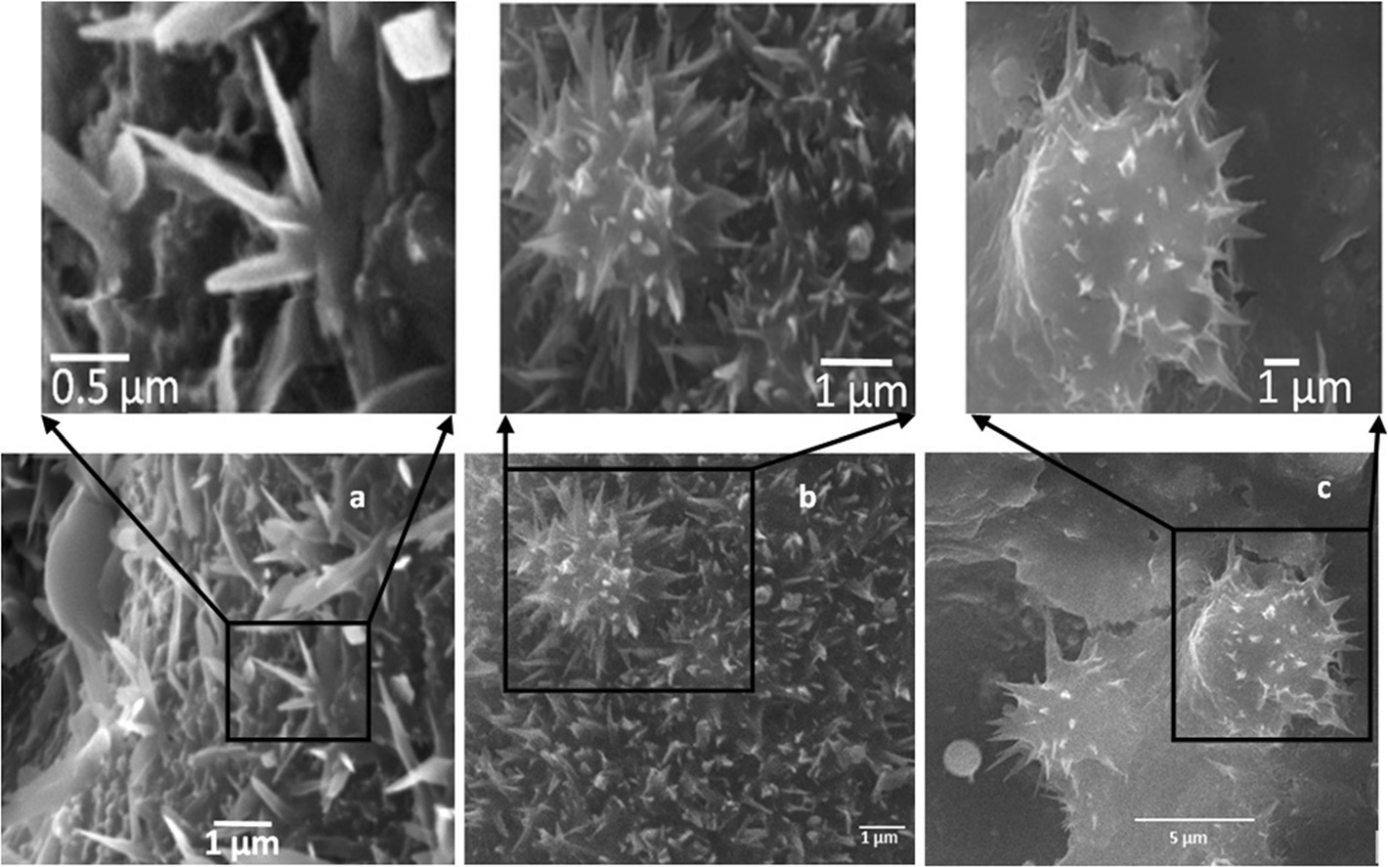
**Nanotip growth induced using different pulse-width sizes under the same laser conditions.** SEM images of nanotips grown on the target surface irradiated with (**a**) 214-, (**b**) 428-, and (**c**) 714-fs laser pulses at 0.5-ms dwell time and 16-W average laser power.

Figure 4 shows SEM images of the randomly selected spots from the irradiated target surface with 214-fs laser pulses. As can be seen, the individual and flower-like nanotips are found to be growing on the target surface. A very interesting pattern is observed in terms of the type of nanotips grown according to the pulse width. When the laser pulse width was increased from 214 to 428 fs and 714 fs, only the nanotips formed from the film of molten target material or large droplets were found to be growing on the target, as observed in Figure 5. The formation of such different types of nanotips can be understood by considering the investigation conducted by Breitling et al. on the vapor flow analysis of the plasma created on the aluminum target under ambient atmosphere [22]. Their study revealed that the vapor-plasma expansion is much more like regular mushroom cloud for longer pulses, whereas it is more turbulent for the shorter pulses. This is mainly due to the disturbances caused using much longer propagation length and by nonlinear radiation-gas interactions for short pulses [22].

In our study, the nitrogen gas flow generates extra turbulence in expanding the plasma. As a result, the plasma species experience many collisions with each other, resulting in the formation of larger droplets. The longer pulse creates high temperature in the target surface, resulting in most of the redeposited droplets being spread into the film before getting cooled down into their original shape using nitrogen gas. There are still chances of forming smaller droplets in the plasma vaporization since plasma species interaction is very random. However, the smaller droplets are most likely to get dissolved into the surface molten layer because of the higher target surface and molten film temperatures. At 428-fs pulse width, as seen in Figure 5b, there are a significant number of nanotips growing from the molten film. When the laser pulse width was further increased to 714 fs, a very small number of nanotips are found to be growing even though it formed from the molten target material, as observed in Figure 5c. This might be due to the fact that during the 714-fs pulse interaction with the target surface, a very large amount of molten material is created which gets ejected into the plasma as well as pushed around the drilled hole due to the shock waves in the plasma. As a result, very short nanotips are observed to be growing from relatively large liquid volume of molten glass, as seen in Figure 5c.

### Effect of laser pulse repetition rate

We have studied three different pulse repetition rates (13, 8, and 4 MHz) in our experiments. As the repetition rate is reduced, the time separation between two pulses increases which eventually changes how the pulse energy is being transferred into the target and being used to ablate the material from the target. If the time gap between two pulses is less than the time required for heat to diffuse out of the focal volume for a typical glass, then the heat will accumulate from the subsequent pulses in the focal volume and elevate the target temperature on the surface and in the bulk. The characteristic thermal diffusion time in glass is about 1 μs for a volume of 0.3 μm^3^[23]. This thermal diffusion time will vary from glass-to-glass according to their composition. However for this report, we are taking this value as a reference. In comparison to this thermal diffusion time, the separation time between two pulses is much smaller; 77, 125, and 250 ns for 13-, 8-, and 4-MHz repetition rates, respectively. Even though all the aforementioned times are much less than the heat diffusion time of 1 μs, the heat accumulation will be high in and around the focal volume at higher repetition rate compared to lower repetition rate. As a result, the energy per pulse required to start the breakdown reduces as the pulse repetition rate is increased. This breakdown threshold energy per pulse is found to be 2.032, 1.338, and 0.862 μJ for 4, 8, and 13 MHz, respectively.

**Figure 6:**
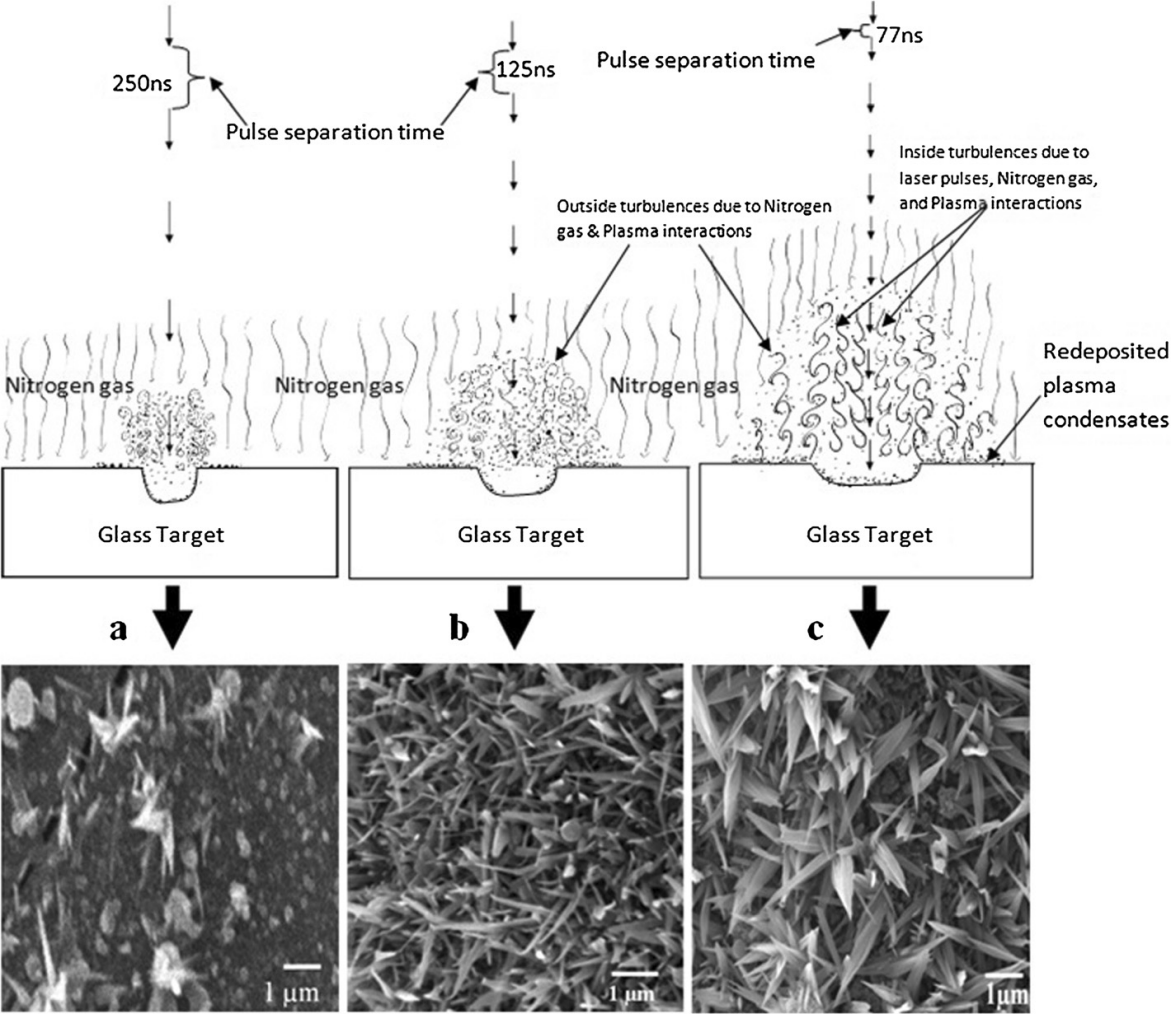
**Effect of laser pulse repetition rate on plasma expansion and nanotip growth.** Nanotips generated for the average laser power of 16 W for pulse repetition rates of (**a**) 4, (**b**) 8, and (**c**) 13 MHz; the dwell time was 0.5 ms.

As the repetition rate is decreased, the size of the tips and the number of tips grown varies. These changes in nanostructure can be explained by how the incoming laser pulses interact with target and the plume of ablated species for each repetition rate. High repetition rates provide more pulses hitting the same spot for a given dwell time in comparison to lower repetition rates. In our investigation, the dwell time is 0.5 ms which provided 6,500, 4,000, and 2,000 pulses for repetition rates of 13, 8, and 4 MHz, respectively. The laser power used was on average 16-W which provides the pulse energies of 4.00, 2.00, and 1.23 μJ for 4-, 8-, and 13-MHz repetition rates, respectively. Although the pulse energy (1.23 μJ) and the pulse separation time (77 ns) between two subsequent pulses, as mentioned above, have the smallest value, the heat build-up is the highest for 13-MHz repetition rate in comparison to other two repetition rates. The reason for this is that the plasma created by the previous pulse does not have enough time to relax before the subsequent pulse arrives in the focal region which further heats the plasma species. As a result, for each progressive number of pulses, a much larger volume than the focal volume is heated above the melting temperature of the glass and larger diameter, compared to laser beam spot diameter, of glass melts on the surface due to highly heated plasma and interaction of the laser pulses [23]. Thus, the plume generated at higher repetition rate is much wider and lasts in air for a longer time, as depicted in schematics of Figure 6c. At a higher number of pulse interaction, the vapor distribution inside the plume rapidly loses its symmetry and becomes more and more turbulent [22]. The turbulences at the outer edges of the plasma are generated due to the interactions between incoming nitrogen gas flow and the outgoing plasma species. The turbulence in the core of the plasma results due to the interactions between the highly energized plasma species due to incoming laser pulse absorption and nitrogen gas molecules. Due to the more turbulent interactions and excessive plasma material during 13-MHz repetition rate machining, the plasma species expand wider, and thus, the redeposition back to the target surface occurs over a larger surface area resulting in the formation of a much larger number of randomly oriented leaf-like nanotips, as seen in Figure 6c. When the ablation is performed at the 8-MHz repetition rate, the plasma must have ideal condition in terms of the amount of the turbulence and available ablated material resulting in the growth of highly populated and oriented narrower nanotips compared to 13 MHz, as seen in Figure 6b. For a low number of pulses, the plasma expansion and interaction with surrounding nitrogen gas is less turbulent. The plasma has more time to relax before the next pulse arrives. Thus, the plasma does not expand outward as much resulting in the plasma species being closer. This resulted in the formation of larger droplets of vapor content which get deposited over the target surface area. As a consequence, only a few nanotips are found to be growing randomly from large droplets for the 4-MHz repetition rate, as seen from Figure 6a.

### Effect of dwell time

**Figure 7:**
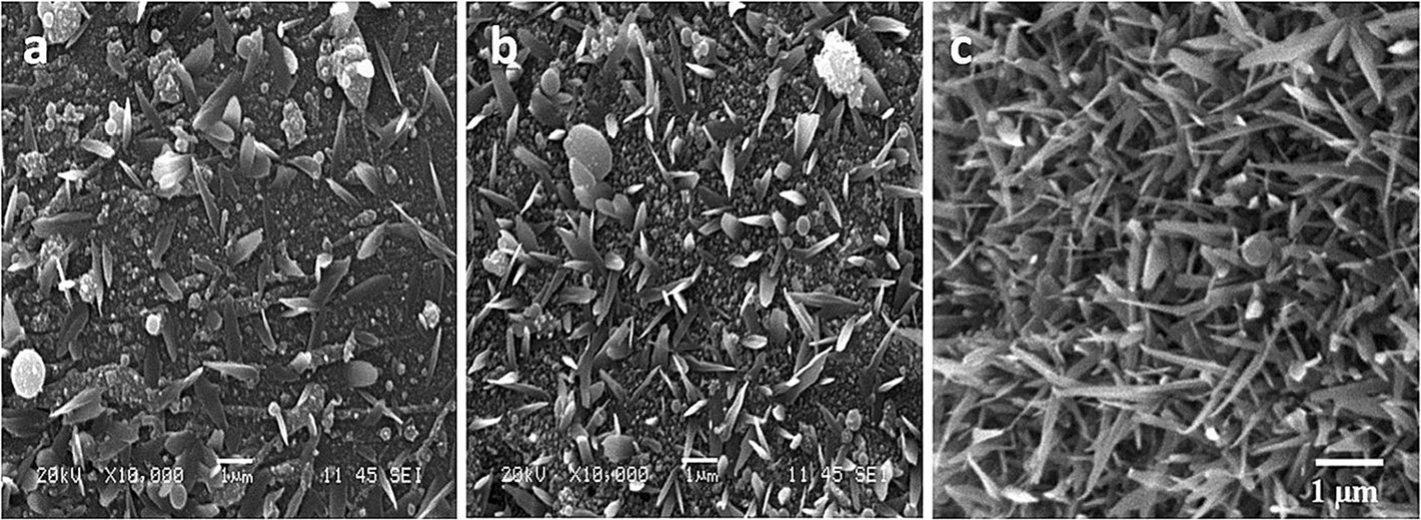
**Nanotip growth under different femtosecond laser irradiation dwell times.** SEM images of nanotip growth stages at the 8-MHz repetition rate for dwell times of (**a**) 0.1, (**b**) 0.25, and (**c**) 0.50 ms for 214 fs and 16-W average laser power.

The dwell time study was performed for 214-fs pulse width and various repetition rates. Figure 7 shows the SEM images of the glass target machined at dwell times of 0.1, 0.25, and 0.5 ms for the 8-MHz repetition rate. The growth steps of the nanotips are clearly evident from these three images. As a result, it is obvious from Figure 7 that the growth of these nanostructures is dependent on the dwell time as much as on other laser parameters. For example, at 0.1 ms, the plasma has very little vapor content resulting in the redeposition of the droplets on the target surface and the growth of stem for the nanotips, as seen in Figure 7a. Once the stem growth has started, the continuous redeposition of vapor condensates from plasma back to the surface provides the building material for tips to grow. At 0.25-ms dwell time, the plasma has just enough building material for the tips to start growing in a nanoscale to a micrometer length; the number of tips present on surface also increased. When the dwell time is further increased to 0.50 ms, the nanoscale tips grew to the length of 1 to 2 μm as well as their population increased on the target surface.

**Figure 8:**
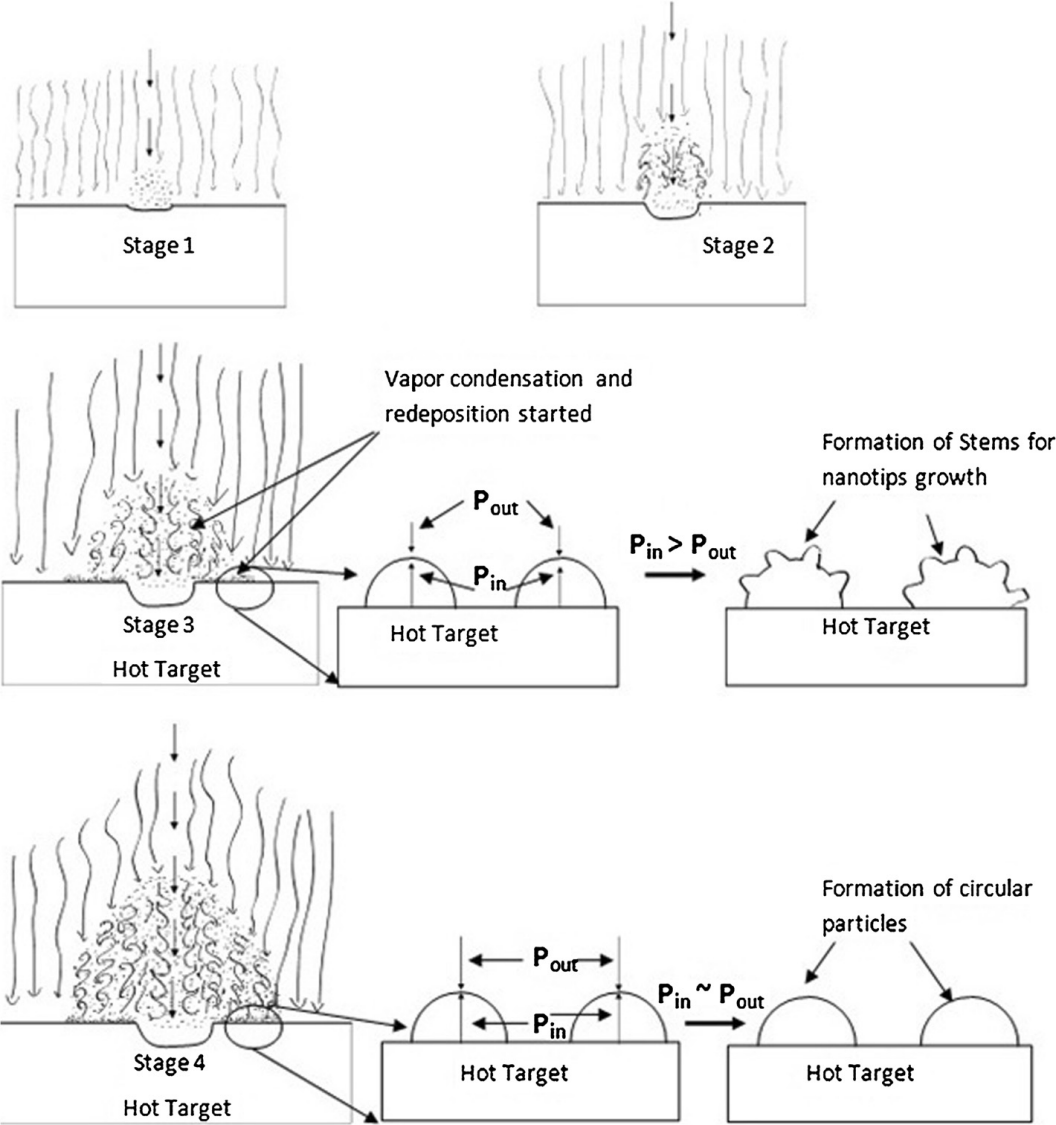
Schematic representation of the growth stages of plasma expansion and nanotips' stem formation.

The repetition rate and dwell time affect the growth of nanotips in somewhat similar way since both control the number of laser pulses delivered to the target surface. After the breakdown of the target material has started, it requires a certain number of pulses according to the repetition rate and dwell time to ablate the required amount of target material into the plasma, as demonstrated in stages 1 to 3 in Figure 8. Before this point in time, the plume does not have enough monomers to start vapor condensation. Once the vapor condensation has started inside the plume, the vapor condensates begin to get deposited onto the hot target surface, as depicted in stage 3. If the machining is stopped little after reaching stage 3, there will not be any more incoming pulses that transfer energy to the plasma species to generate further turbulence. As a result, the plasma species start relaxing by cooling down and mixing with nitrogen gas molecules. The consequence of these phenomena will be that the pressure exerted due to the plasma species will be relieved and the internal pressure becomes much higher than the external pressure on the deposited plasma condensates due to the hot target surface. At the same time, the deposited condensates experience uneven cooling due to the random flow of nitrogen gas. As a result, the deposited droplets have regions of high and low surface tension over their entire surface. As a result, the imbalance of pressure pushes the material out of the volume of deposited droplets from regions of low surface tension resulting in the formation of the stem for the nanotips, as depicted in the side figures of stage 3 in Figure 8.

**Figure 9:**
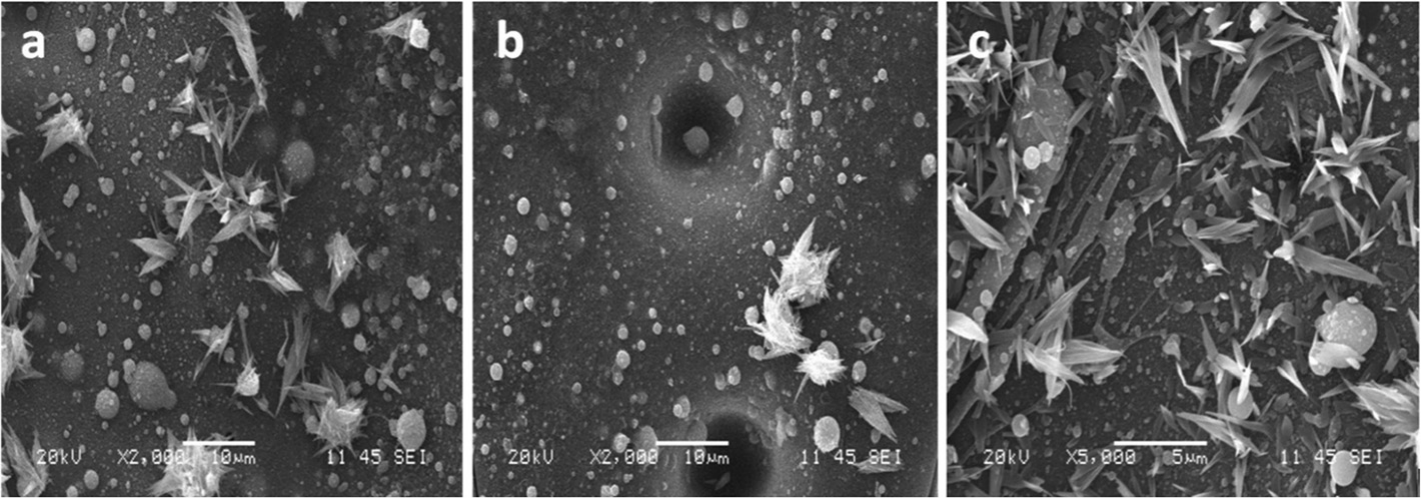
**Effect of excessive machining of irradiation spot corresponding to various repetition rates.** Nanostructures generated at the dwell time of 0.75 ms for the repetition rates of (**a**) 4, (**b**) 8, and (**c**) 13 MHz for 214 fs.

The ablation mechanism somewhat changes from one repetition rate to another due to the difference in threshold energy-per-pulse requirement. The incoming pulses also interact differently with plasma generated from previous pulses for each repetition rate. Thus, the nanostructures generated for even the same dwell time differ for different repetition rates, as seen in Figures 6 and 9. Figure 9 shows SEM images of the glass target irradiated with 4-, 8-, and 13-MHz repetition rates for a dwell time of 0.75 ms. For 8- and 13-MHz repetition rates, the number of nanotips produced is much less compared to 0.50 ms, as seen in Figures 6 and 7. Instead, the presence of many spherical micronanoparticles and molten droplets is observed. This phenomenon can better be understood from the stage 4 of the schematic representation depicted in Figure 8. When the irradiated spot is bombarded with too many pulses as in the case of high repetition rates and high dwell time, an excessive amount of material is added to the plasma. The incoming subsequent pulses also interact with the plasma species elevating their temperature and giving them high kinetic energy. As a result, the plasma expands outward faster and to the larger radius exerting more pressure in the surrounding including onto the redeposited plasma vapor condensates on the target surface. This creates the external pressure approximately similar to or higher than the internal pressure of the redeposited material, hence hindering the formation of stems, stage 4 of Figure 8. The excessive temperature of the plasma species and the target can also remelt the deposited material as well as previously grown stems and tips. The SEM image of the target irradiated with 13-MHz repetition rate for the dwell time of 0.75 ms depicted in Figure 9c is the perfect example of the stage 4 illustrated in Figure 8. For 8-MHz repetition rate at 0.75-ms dwell time, most of the redeposited material must be experiencing approximately equal internal and external pressure resulting in the formation of just circular micronanoparticles rather than the formation of stems. There is an evident of the formation of very few tips from bulk droplets in Figure 9b. If we follow the above four stages, there should not be any tip growth for 13-MHz repetition rate for the dwell time of 0.75 ms. However from Figure 9c, it can be seen that a significant number of nanotips grew on the target. This happened because the 13-MHz repetition rate provides a much larger number of pulses and the machining is performed way beyond stage 4 of the growth mechanism. When the plasma reaches stage 4, it will exert excessive pressure and temperature on previously deposited material resulting in remelting and formation of micronanoparticles. But at the same time, since plasma is continuously being heated by incoming pulses, plasma will rapidly expand outward. There will be a point in time where the plasma has expanded far enough from the redeposition site relieving excessive pressure and temperature. From this point onward, the transmission of the subsequent laser pulses will improve, and the new material will be ablated from the target forming new plasma over the target surface. This whole phenomenon must be occurring in the last part of the 0.75-ms dwell time during which the growth mechanism starts back at stage 1 and forms nanotips on previously deposited material, as seen in Figure 9c.

### Effect of laser polarization

All the experiments discussed above were performed by circular polarization of femtosecond laser pulses. We also wanted to investigate whether the linear polarization changes the growth mechanism of nanostructures on the laser-irradiated target glass. The effect of laser polarization on the ablation of various materials has been studied by many researchers. Hee et al. studied the effect of polarized femtosecond laser pulses on the generation of relief gratings on (111) silicon substrate using a novel interferometer [24]. The ablation was performed by focusing two interfering femtosecond laser beams under different polarization combinations. In their investigation, they found that p:-p-polarization has the lowest ablation threshold and generates the deepest grating depth among other polarization combinations (s-:s-polarization; c-:c-polarization). Camacho-Lopez et al. investigated the growth of grating-like structures on titanium films by circular (c-) and linear (p-) polarizations [25]. They discovered that there was no formation of grating-like structures when the substrate was irradiated with circularly polarized light. However, when linearly polarized laser pulses were utilized, the grating-like structures were generated at the fluence well below the ablation threshold for the titanium film. Furthermore, Venkatakrishnan et al. also found in their study of polarization effects on ultrashort-pulsed laser ablation of thin metal films that linear (p-) polarization has an ablation threshold less than that for circular polarization [26].

**Figure 10:**
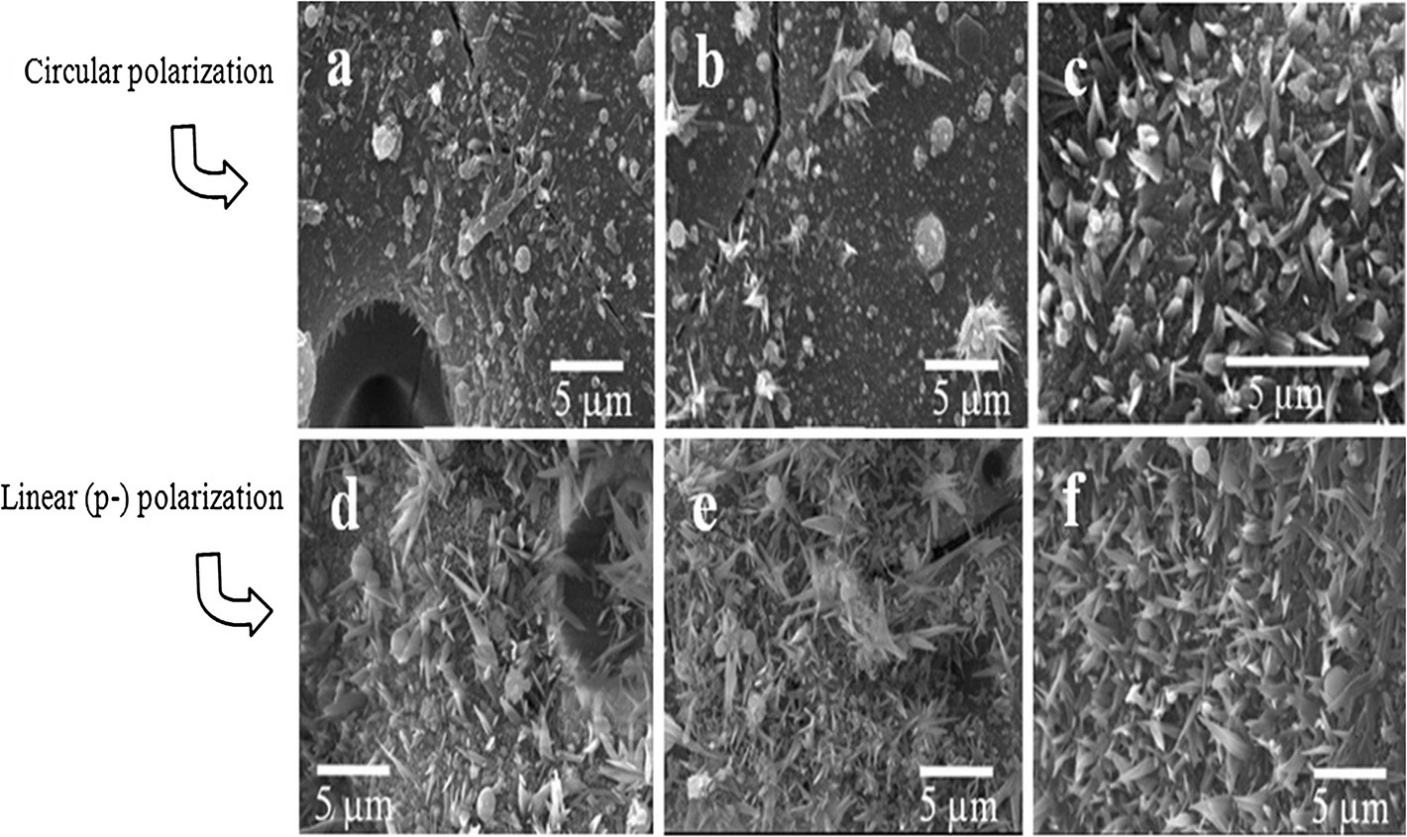
**Comparison of nanotip growth under different polarizations of laser pulses.** SEM images of the glass target irradiated with circularly polarized pulses (**a**, **b**, **c**) and linearly (p-) polarized laser pulses (**d**, **e**, **f**); (**a**, **d**) 4 MHz, 0.25 ms; (**b**, **e**) 4 MHz, 0.5 ms; (**c**, **f**) 8 MHz, 0.25 ms; the pulse width used for all experiments was 214 fs.

In our investigation, we found results that support the findings in the aforementioned investigation performed by other researchers. We found that when the glass was irradiated by p-polarized laser pulses, a much larger number of nanotips were found to be growing for the same parameters in comparison to circularly polarized pulses, as depicted in Figure 10. It was found by other researchers that the p-polarized laser pulses ablate the target material at fluences much smaller than the ablation threshold fluence for circular polarization. If this is true, then the p-polarized pulses remove material much more efficiently with much fewer pulses in comparison to circularly polarized laser pulses. In other words, the growth stages explained in Figure 8 must be occurring in the fast-forwarding mode during linearly polarized laser ablation.

Looking at the SEM images in Figure 10, these changes can be better understood. Figure 10a shows the SEM image of the target irradiated with circularly polarized laser pulses with 4-MHz repetition rate at the dwell time of 0.25 ms. It can be seen that there is no evident of tip growth most likely due to the inadequate ablated material into the plasma. When the target was irradiated with linearly (p-) polarized pulses with the same laser parameters, as depicted in Figure 10d, a high number of nanotips were found to be growing on the target surface. This is only possible if the linearly polarized pulses ablated an adequate amount of material from the target into the plasma in order for the nanotips to initiate and complete their growth. To further make sure if this is the case for other laser parameters with linear polarization, we also irradiated targets at 0.5-ms dwell time for 4 MHz and at 0.25 ms for 8 MHz. The corresponding SEM images of these experiments are shown in Figure 10. For each parameter, it was found that the growth of nanotips improved in terms of density of nanotips over large target surface at each parameter. From this result, it can be understood that the linear (p-) polarization does not really alter the nanotip growth mechanism but rather it enhances it. Since linearly polarized pulses ablate material more effectively even at the same pulse energy in comparison to circular polarization, it will take fewer numbers of pulses while using linear polarization to reach each growth stage explained in Figure 8. Now that we know how the growth of nanotips is affected using various femtosecond laser parameters, it will be beneficial to perform *in situ* analysis of the plasma expansion, the process temperature, and pressure gradient for each combination of the laser parameters. This future work will help us find out the exact combination of femtosecond laser parameters which will produce more uniform and maximum number of nanotips over the large surface of the dielectric targets.

## Conclusions

In summary, we have discussed the growth of leaf-like nanostructures with nanoscale apex from dielectric target material by femtosecond laser irradiation at megahertz pulse repetition rates. In our synthesis method, the whole growth process occurs in an open air at ambient conditions in the presence of nitrogen gas flow without the use of any catalyst. The dielectric target provides two roles: first as the source for building material and second as the substrate upon which these leaf-like nanotips can grow.

The growth mechanism of nanotips is explained by classic thermal diffusion. We observed the growth of individual and multiple nanotips from relatively small single droplets at shorter pulse width; whereas when the pulse width was increased, the nanotips grew mainly from the film of the molten target material and the large deposited droplets of molten material.

The laser specifications (laser pulse width, pulse repetition rate, and laser polarization), processing parameters (dwell time), and gas flow rate control the number of tips synthesized and, to some extent, the size of tips. In our investigation, we found the clear transformation of the kind of nanotips that grow under various conditions. In further experiments, we found that for a given dwell time, the number of nanotips that grow on target surface increases with increasing pulse repetition rate. However, this was only observed for certain dwell times. If the laser machining was continued on a spot beyond a certain dwell time for each repetition rate, we observed fewer nanotips grown on the target along with the growth of increasing number of micronanoparticles and remelting of previously deposited plasma material. The dwell time was observed to be influencing the nanotip growth in a similar manner as pulse repetition rate; at low dwell time, only the growth of a small number of stems was observed. As the dwell time was increased for a given repetition rate, an increasing number of stems and nanotips were found to be growing on the irradiated target surface. Finally, we studied the effect of linear polarization on the growth of leaf-like nanotips. We observed the enhanced number of nanotips grown on the target surface in comparison to machining under circular polarization of the laser for the same given laser parameters.

Future work will involve the *in situ* analysis of plasma interactions with nitrogen gas flow and incoming laser pulses, the pressure and the temperature gradient of target surface, and the expanding plasma. Understanding the aforementioned phenomena *in situ* will provide more control and help us grow more uniform nanotips over the large surface area of the target. This study was carried out with silicon substrate, but we believe that other semiconductor materials may also generate similar phenomena.

## Competing interests

The authors declare that they have no competing interests.

## Authors’ contributions

NP designed the experiment, collected experimental results, and involved in analysis and interpretation of data. He was the person in charge of drafting this manuscript. KV created the concept of using femtosecond laser for nanotips synthesis. He has made substantial contributions to the acquisition of data, and analysis and interpretation of data. BT made substantial contributions to the acquisition of data, and analysis and interpretation of data. She has been involved in drafting the manuscript and revising it critically for important intellectual content and has given final approval of the version to be published. All authors read and approved the final manuscript.

## Authors’ information

NP was a candidate of Master of Applied Science. KV is the co-supervisor of NP. BT is the supervisor of NP.
